# MagicCubePose, A more comprehensive 6D pose estimation network

**DOI:** 10.1038/s41598-023-32936-3

**Published:** 2023-04-28

**Authors:** Fudong Li, Dongyang Gao, Qiang Huang, Wei Li, Yuequan Yang

**Affiliations:** College of Information Engineering (Artificial Intelligence College), Yangzhou University, Yangzhou, 225000 Jiangsu China

**Keywords:** Computational science, Computer science

## Abstract

Most of the current mainstream 6D pose estimation methods use template or voting-based methods. Such methods are usually multi-stage or have multiple assumptions and post-correction, which will cause a certain degree of information redundancy and increase the computational cost, their real-time detection performance is poor. We point out that traditional path aggregation networks introduce new errors, therefore, we propose a loss function: MagicCubeLoss, a portable module: MagicCubeNet, and the corresponding 6D pose estimation model: MagicCubePose. MagicCubePose has good expansion performance and can build more efficient models for different calculation power and scenarios. Experiments show that our model has good real-time detection performance and the highest ADD(-S) accuracy.

## Introduction

Since its appearance in the 1960s, machine vision has made great strides in many fields. With the in-depth research and application of deep learning, traditional 2D object location and recognition methods have been unable to meet the needs of social development, so some scholars try to study 3D object detection and 6D pose estimation based on deep learning methods. 6D pose estimation has a wide range of application scenarios, such as the self-driving cars, augmented reality, robotics and other application fields which have high requirements of spatial positioning information and the complexity of the scene of the detected target^1,2^ .

Although deep learning has certain advantages in dealing with the above problems, different models have different problems in dealing with different application scenarios. The method of obtaining RGB-D image data through a depth camera has good robustness^3^, but the data processing of images with depth information is far more complicated and computationally expensive than ordinary RGB images. High-quality depth cameras are expensive and not portable, it is not friendly to real-time detection tasks in some mobile scenarios^4,5^. With the in-depth research of deep learning, the method based on RGB images is as robust as the RGB-D method.

SSD-6D^6^ proposes a new SSD^7^-based method to detect 3D model instances and perform 6D pose estimation directly from RGB data, which verifies that the model with RGB data is better than other models with RGB-D data. But for the most difficult detection sequences, such as “camera, milk” with serious occlusions, they still have the problem of missed detection and low detection effect. Poor detection performance for smaller objects, possibly due to the presence of blind spots or their lack of texture and uniform color, making them indistinguishable from the environment. Although the one-stage structure design is fast enough, the precision is not ideal. Through its structure design and core algorithm idea, we know that the method of 6D pose prediction with key points is not effective in complex scenes such as occlusion and poor target texture. It is a common situation especially in lower resolution video stream.

In this paper, a new 6D pose detection model is proposed, which still adopts a one-stage structure design, taking RGB images as input, realizing end-to-end training and directly detects the 2D projection of 3D bounding box, even without post-processing of poses. It also has a good accuracy rate. Besides eliminating the post-processing step, our method does not build a textured 3D model like other template-based methods to increase the pre-training workload and computational cost.

We perform validation tests on the LINEMOD^8^ dataset, which has become the standard benchmark for 6D pose estimation. Compared with YOLO6D^4^, which is 5 times faster than other methods when dealing with single object, our method does not lose to it, when performing multi-object detection, our method has higher accuracy.

To summarize, the main contributions of our work are: based on the EfficientDet^9^ network structure, we design a new loss function called MagicCubeloss and the corresponding pose estimation model: MagicCubePose, which can effectively reduce the deflection error introduced during data augmentation, and it does not require pose refinement, it can realize fast and high-precision 6D pose measurement.

## Related work

We review common RGB image-based 6D pose estimation methods, ranging from template-based to voting-based methods.

### Templete-based

PoseCNN^10^ proposes a new pose dataset YCB^10^, which estimates the 3D displacement by locating the center of the object in the image and estimating its distance from the camera, and obtains the 3D rotation by regressing to the four-element^11^ representation, a new loss function is proposed to enable it to recognize symmetric objects: two loss functions are used for the object symmetry (shapematch-loss) and the asymmetry (pose-loss) train. Pix2Pose^12^ adopts a two-stage network structure design similar to PoseCNN. First, mask prediction and bounding box positioning are performed, and then pixel-level 3D coordinate regression is performed. The 3D coordinates of each pixel of the object can be directly predicted without the need for accurate texture 3D models. A new loss function is proposed to deal with the pose problem of symmetric objects. HybridPose^13^ uses hybrid intermediate representations to express geometric information (key points, symmetric correspondences, edge vectors), does not use depth information, utilizes semantic edge vectors of adjacent edge key points, three-stage intermediate representation method: key points , edge and symmetry corresponds, the key point is the main, edge and symmetry are the auxiliary.

The template-based methods can better deal with the object pose problem with poor texture. First, the 3D model of the target is established to obtain its templates from different perspectives, and then the best match (the pose of the best template) is obtained by calculating the similarity scores of different positions. However, it does not perform well when dealing with occluded object poses.

### Voting-based

PVNet^14^ regresses the pixel unit vector to obtain key points, and uses RANSAC^15^ to vote to obtain key point positions. Although RANSAC-based voting solves discrete point prediction and gives the spatial probability distribution of key points, the voting method produces uncertain key points allow the pnp algorithm^16,17^ to better predict the final pose, but the traditional two-stage approach (locating key points first, then solving the pose by pnp) only locates sparse key points, as for the occluded objects, they cannot fully express their characteristics.

A Hybrid Approach for 6DoF Pose Estimation^18^ firstly segment the target instance and then restore it to 6D pose by point-to-point voting, automatically select the best-performing instance detector and training set, thanks to the CNN structure design filtering highly unstructured data and successfully used in complex scenarios.

### Correspondence-based

BB8^19^ uses a CNN network for the first time to predict the 3D pose of an object directly through the 2D projection of 3D bounding box, and provides an extra step to optimize the predicted pose. Many objects in the T-LESS data are (semi) symmetric, which means that different poses may have similar results, which makes CNN training more difficult, limits the range of poses used for training, and introduces a classifier to identify poses range during training and then perform pose estimation.

YOLO6D is a real-time single-shot 6D pose estimation model with superior performance, based on YOLO^20–23^. YOLO6D uses the CNN structure to directly predict the 2D projection of the 3D bounding box vertices, and then directly returns to the 6D pose through the pnp algorithm without post-processing. It is significantly better than other recent CNN-based methods for post-processing. Other methods are much slower than YOLO6D, but YOLO6D does not involve occlusion and symmetrical object detection.

The above methods all return to the 6D pose through 2D projection: first locate the 2D target position, then obtain the relevant parameters of the 3D bounding box, and finally return to the 6D pose. A common data augmentation method is to render the object into a randomly selected background image from the COCO^24^ dataset. However, BB8 performs semantic segmentation on the target and predicts 8 2D corners in the second stage of the network, and the other two methods are direct regression. SSD-6D performs optimal screening through NMS. 2D bounding box obtains possible viewpoints and plane rotations and establishes a 6D pose hypothesis set. Discrete viewpoints (combined with plane rotation) can effectively solve the problem of low pose estimation of symmetrical or occluded objects. BB8 introduces A classifier to solve the problem of symmetric object rotation.

## Method

Before formally introducing our method, we briefly summarize the previous work. We summarize all 6D pose estimation methods into two categories: building a 6D pose estimation model directly and building a 6D pose estimation model based on the extension of the 2D object detection network.

In previous chapters, we introduce partial pose estimation networks from template-based to voting-based methods, Ref.^10–14^ build 6D pose estimation models directly, and we found that this method has a clean network structure and high detection accuracy, but the corresponding real-time performance is relatively poor and the model expansion performance is low. Ref.^4,6,27^ build 6D pose estimation network by extending 2D object detection model. The backbone network of this type of model is relatively simple, but they have better scalability and real-time detection performance.

To sum up, we finally decided to use the method of constructing a 6D pose estimation model based on the extension of the 2D target detection network to verify our method. Considering the needs of different scenarios and calculation power, we will optimize the extension network based on EfficientDet^9^ design.

### Overall Model Architecture

**Figure 1:**
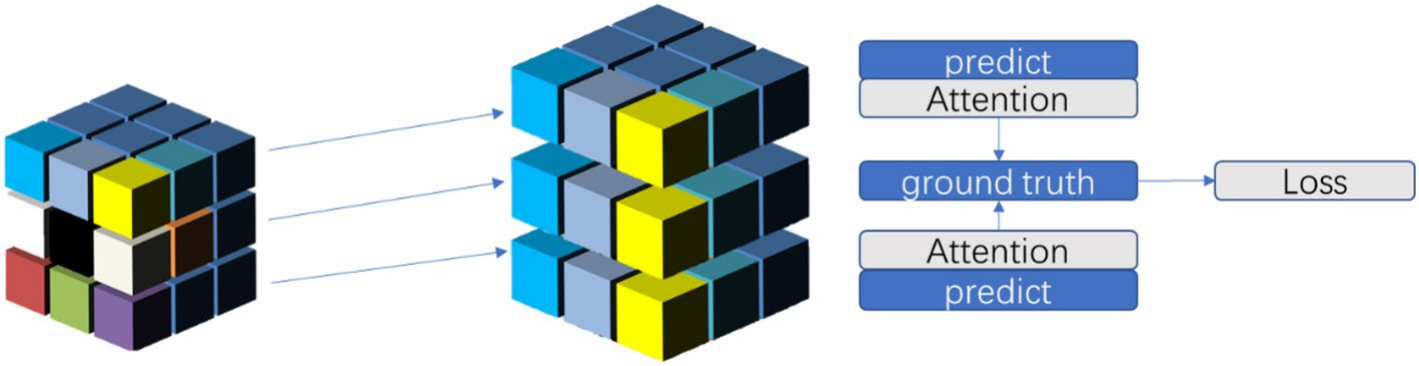
The architecture of MagicCubeNet.

**Figure 2:**
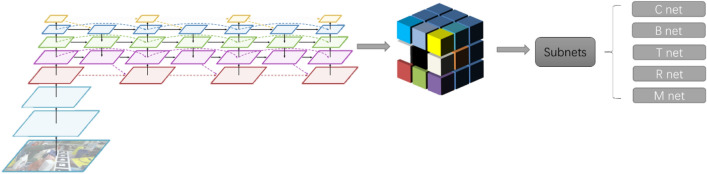
The architecture of MagicCubePose, which is based on EfficientDet, including the EfficientNet backbone, the bidirectional feature pyramid network (BiFPN) and MagicCubeNet.

Inspired by the structure of the MagicCube, we design a network structure module named MagicCubeNet, as shown in Fig. 1, using EfficientNet as the backbone network, as shown in Fig. 2, and we also reference EfficientPose^27^ to build a network of pose estimation module and expand and optimize it, finally the pose estimation network design of MagicCubePose is realized.

When we study the path aggregation network and the BiFPN network, we find that their core design ideas are very similar, both of which obtain feature maps with richer semantic information through multiple convolutions of horizontal and vertical graphs of convolutional layers. If we take the top-level feature map of the complete BiFPN as an example (yellow feature map shown in Fig. 2), from left to right named FM_1, FM_2, FM_3, FM_4 respectively, after multiple feature fusion, despite the same shape, the semantic information are different from each other, but the difference is controlled in a small range. In other words, it is not worth the model to spend so many resources to compose a feature map that does not directly affect the final detection accuracy. We refer to this behavior as cost error. In order to effectively solve this error, combining the design of MagicCubeNet and Smooth L1 loss^24^, we finally obtained MagicCubePose.

### Regress 6D pose

Taking the third-order magic cube as an example, we abstract the single-layer feature map extracted from the BiFPN backbone structure into the middle layer of the magic cube, and then randomly select two features in the BiFPN extended network which are at the same level as the above middle layer. Considering that the process of model reproduction is time-consuming, and our method is not very demanding on the handware conditions of the device, to validate our methods quickly and efficiently, we only build, train and verify based on the sub-top level feature map and its extended structure in the BiFPN backbone network.

Different from the previous loss design which calculate the difference between the prediction and ground truth one time in each iteration. As shown in Fig. 1, after completing the most basic structure, the first layer and third layer of the magic cube are marked as P1 and P3 as the predict layer, the second layer as the ground truth layer is marked as P2. We also introduce the attention mechanism^25^, after that we get P1_A and P3_A, here, we get two set of data: P1_A &P2, P3_A &P2, then we calculate their loss through smooth L1^24^ and finally get the whole loss.

Here we directly imitate the method in^27^ to construct a sub-network from 2D object to 6D pose estimation, which includes: Class(C net)

Bbox(B net)

Trans(T net)

Rotation(R net)

These four parts build the final loss of^27^:

1 $$\begin{aligned} L = \lambda _{class} \cdot L_{class} + \lambda _{bbox} \cdot L_{bbox} + \lambda _{trans} \cdot L_{trans} \end{aligned}$$

Based on the structural design of MagicCubeNet, we newly added Mnet, which is the Loss in MagicCubeNet: MagicCubeLoss.

Compared with L1 and L2, Smooth L1 converges faster and insensitive to outliers, the gradient change is relatively small, and it is not easy to cause gradient explosion during training.

2 $$\begin{aligned} L_{M}=smooth L1 = {\left\{ \begin{array}{ll} 0.5x^2, \mid x \mid \leqslant 1\\ \mid x \mid - 0.5, \mid x \mid > 1 \end{array}\right. } \end{aligned}$$

So, the final MagicCubeLoss function is as follows:

3 $$\begin{aligned} L = \lambda _{class} \cdot L_{class} + \lambda _{bbox} \cdot L_{bbox} + \lambda _{trans} \cdot L_{trans} + \lambda _{M} \cdot L_{M} \end{aligned}$$

Among them, trans is the combined calculation item of translation and rotation, and M is the calculation item of loss in MagicCubeNet. Among them, $L_{class}$ is the classification loss, $L_{bbox}$ the bounding box loss, and $L_{trans}$ is the conversion loss. In order to balance the impact of this part of the loss in the training process, the $\lambda$ parameter is introduced for each part. In addition, we refer to the final loss in^9^ and^27^, designed and combined with our own experimental results, we find that $\lambda _{class}$, $\lambda _{bbox}$ = 1, $\lambda _{trans}$ = 0.02 and $\lambda _{M}$ = 0.01 achieve the best results. At the same time, inspired by the design of^10^: we consider the symmetry and asymmetry of the object when designing the loss function, and achieve ideal results.

## Eexperiments

The experiments in this paper are based on the Tensorflow2.4.0 framework, cuda11.1, 11400F CPU and RTX3070 GPU.

We use Linemod and Occlusion^28^ data. We train 500 epochs and compare our results with the SOTA methods. Since^9^ has good scalability and extensibility, considering our current experimental conditions, we do not fully evaluate the hyperparameter $\varphi$ which used for controlling the depth or width of the model range from 0 to 7, to verify the effectiveness of our method we only use $\varphi$=0 for single object and $\varphi$ =1 for multi-object.

### Dataset

The Linemod dataset is a widely used dataset for 6D pose estimation, it has 13 classes of objects. For different scenes, only the 6D pose of one object is annotated, although there are still several other types of objects in the same scene. To fully verify the superior performance of our method, we also design experiments on multi-object pose detection.

Occlusion data consists of part of Linemod data to annotate multiple targets in a single scene. These objects are mostly occluded, which also makes their pose estimation more difficult.

### Evaluation metric

We use ADD(-s)^29^ and 2D projection^32^ metrics to evaluate our method.

ADD(-s) metric calculate the average distance between ground truth and predict of rotation R and translation T of each point in the 3D model point set M. Considering the symmetry and asymmetry of the object, its evaluation method is also different. The definition of asymmetric target is as follows:

4 $$\begin{aligned} ADD = \frac{1}{m}\sum _{x \in M}\Vert (Rx+T)-({\tilde{R}}x+{\tilde{T}})\Vert _{2} \end{aligned}$$

The definition of symmetric target is as follows:

5 $$\begin{aligned} ADD-S = \frac{1}{m}\sum _{x_{1} \in M}min_{x_{2}\in M}\Vert (Rx_{1}+T)-({\tilde{R}}x_{2}+{\tilde{T}})\Vert _{2} \end{aligned}$$

The pose estimate is considered correct if the point average distance is less than 10% of the object diameter.

2D projection metric.This metric computes the mean distance between the projections of 3D model points given the estimated and the ground truth pose. A pose is considered as correct if the distance is less than 5 pixels.

### Single object detection

**Figure 3:**
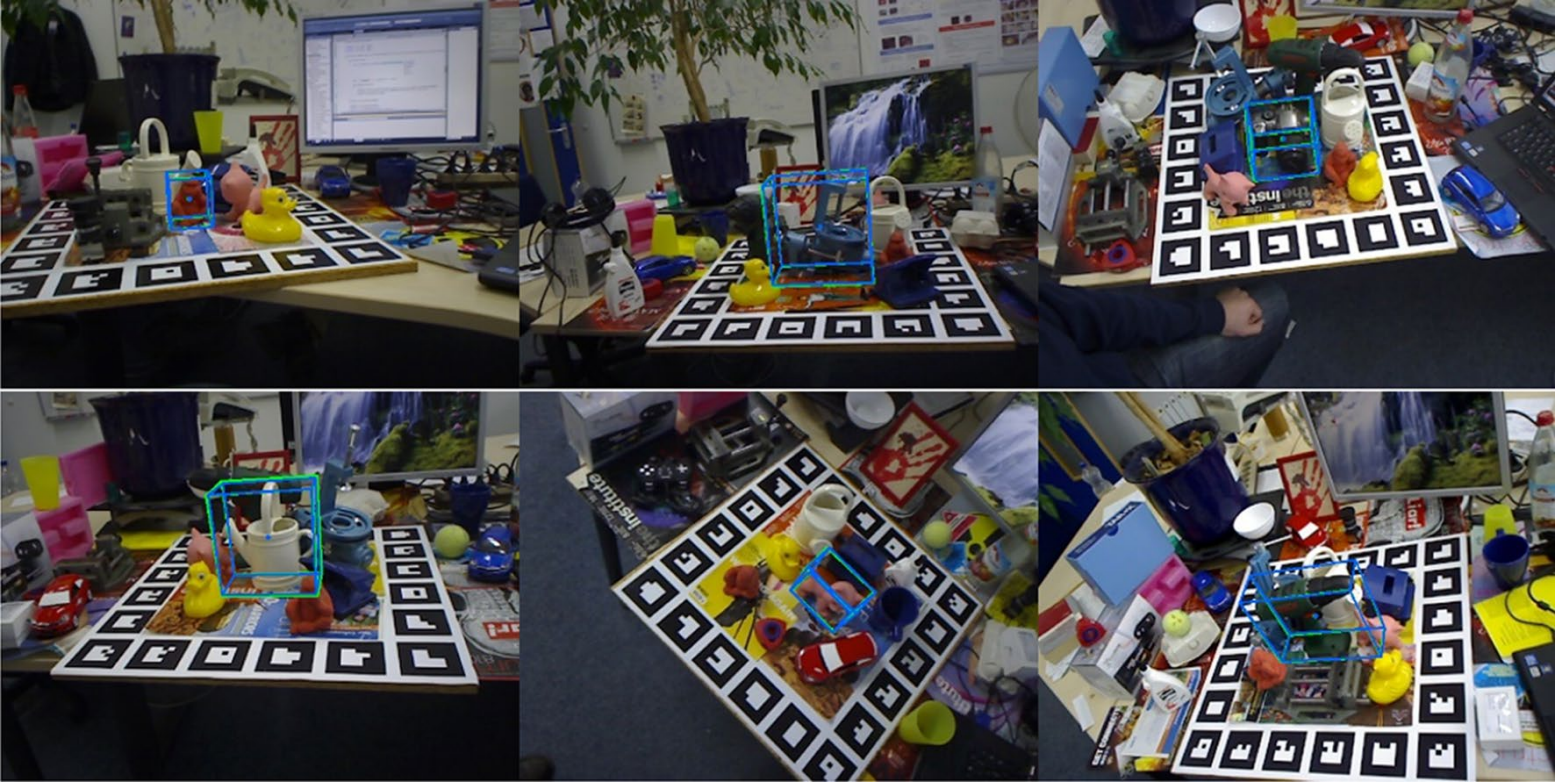
Single object detection map (green is ground truth, blue is predict. The first column is object id: 1 2 4, and the second column is object id: 5 6 8).

**Table 1:** Evaluation and comparison on the Linemod dataset in terms of the ADD(-S) metric. Symmetric objects are marked with * ).

Method	YOLO6D	Pix2Pose	PVNet	DPOD	HybridPose	EfficientPose($\varphi$=0)	RNNPose^31^	Our
Ape	21.62	58.1	43.62	53.28	63.1	87.71	88.19	87.71
Benchvise	81.80	91.0	99.90	95.34	99.9	99.71	100	100
Cam	36.57	60.9	86.86	90.36	90.4	97.94	98.04	98.24
Can	68.80	84.4	95.47	94.10	98.5	98.52	99.31	99.02
Cat	41.82	65.0	79.34	60.38	89.4	98.00	96.41	98.20
Driller	63.51	76.3	96.43	97.72	98.5	99.90	99.70	99.80
Duck	27.23	43,8	52.58	66.01	65.0	90.99	89.30	90.70
Eggbox *	69.58	96.8	99.15	99.72	100	100	99.53	100
Glue *	80.02	79.4	95.66	93.83	98.8	100	99.71	99.90
Holepuncher	42.63	74.8	81.92	65.83	89.7	95.15	97.43	95.62
Iron	74.97	83.4	98.88	99.80	100	99.69	100	99.69
Lamp	71.11	82.0	99.33	88.11	99.5	100	99.81	100
phone	47.74	45.0	92.41	74.24	94.9	97.98	98.39	98.56
Average	55.95	72.4	86.27	82.98	91.3	97.35	97.37	97.50

**Table 2:** Evaluation and comparison on the Linemod dataset in terms of the 2D projection metric. With refinement methods are marked with *.

Method	BB8*	BB8	^34^	Tekin^33^	Our
Ape	96.6	95.3	85.2	92.10	98.76
Benchvise	90.1	80.0	67.9	95.06	97.48
Cam	86.0	80.9	58.7	93.24	98.82
Can	91.2	84.1	70.8	97.44	96.75
Cat	98.8	97.0	84.2	97.41	99.20
Driller	80.9	74.1	73.9	79.41	96.63
Duck	92.2	81.2	73.1	94.65	98.40
Eggbox *	91.0	87.9	83.1	90.33	100
Glue *	92.3	89.0	74.2	96.53	94.20
Holepuncher	95.3	90.5	78.9	92.86	98.29
Iron	84.8	78.9	83.6	82.94	97.65
Lamp	75.8	74.4	64.0	76.87	94.72
phone	85.3	77.7	60.6	86.07	94.81
Average	89.3	83.9	73.7	90.37	97.36

### Multi-object detection

**Figure 4:**
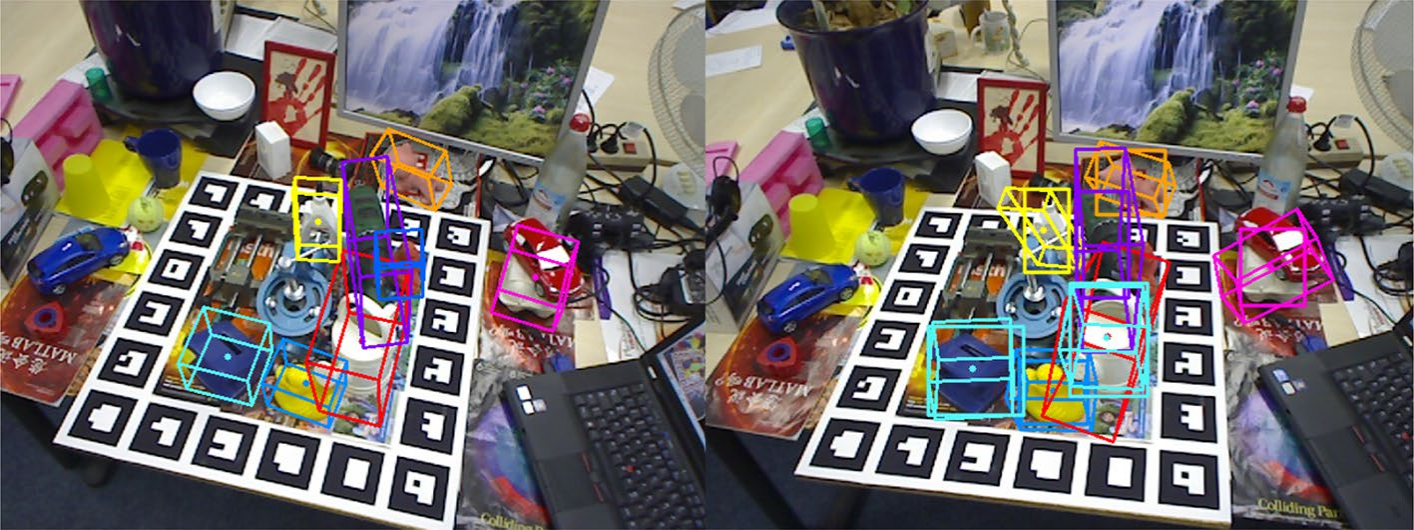
Multi-object(8 objects) detection map (the left picture is 6D pose results, the right picture not only has 6D but also has 2D detection results).

**Table 3:** ADD(-S) metric of multi object 6D pose estimation using a single model on the Occlusion dataset. Symmetric objects are marked with *.

Method	PoseCNN	PVNet	RNNPose	EfficientPose^27^($\varphi$=0)	^27^($\varphi$=3)	Our($\varphi$=0)	Our($\varphi$=1)
Ape	9.60	15.8	37.18	56.57	59.39	56.73	59.34
Can	45.2	63,3	88.07	91.12	93.27	92.21	94.44
Cat	0.93	16.7	29.15	68.58	79.78	68.59	80.13
Driller	41.4	65.7	88.14	95.64	97.77	95.67	97.77
Duck	19.6	25.2	49.17	65.31	72.71	66.54	73.32
Eggbox *	22.0	50.2	66.98	93.46	96.18	95.33	96.34
Glue *	38.5	49.6	63.79	85.15	90.80	85.45	90.81
Holepuncher	22.1	39.7	62.76	76.53	81.95	76.61	81.97
Average	24.9	40.8	60.65	79.04	83.98	79.64	84.27

**Table 4:** End-to-End runtime results.

Method	^9^-D7	PVNet	DPOD	EfficientPose^27^	^27^	^27^	Our	Our
Model($\varphi$)	7	–	–	0	0	2	0	0
Single/Multi	Single	Single	Single	Single	Multi	Single	Single	Multi
GPU	V100	1080TI	TITAN X	2080Ti	2080Ti	3070	3070	3070
FPS	8.2	25	33	27.45	26.22	–	25.65	24.72
Train time(Day)	–	–	–	2.36	–	3.86	2.26	–

**Table 5:** Comparison with state-of-the-art methods on the LINEMOD dataset.

Method	BB8	PoseCNN+DeepIM^35^	Tekin	Faster R-CNN+DeepIM	Our ($\varphi$=0)
5cm5degree	69	85.2	–	83.4	86.64
ADD	62.7	88.6	55.95	86.9	97.5
2D Projection	89.3	97.5	90.37	95.7	97.36

### Analysis

In Table 1, we compare our method with the results of mainstream 6D pose estimation models based on Linemod dataset (not only RGB-based but also RGBD-based methods), and use ADD(-S) evaluation metric. In Table 2, we use 2D projection metric, it is obviously that our method outperforms all currently known methods and requires no further refinement. Even when compared with the SOTA2022 method RNNPose [25], our method is better. Although the detection effect of some objects such as ‘Ape’ and ‘Can’ is slightly lower than it, there are still certain advantages in general. In addition, we also select 6 single targets for detection, and the results are shown in Fig. 3.

However, common object detection tasks are often multi-object, which is undoubtedly a challenging task. To fully verify the performance of our method, we conduct multi-object detection experiments, and the experimental results are shown in Table 3. Compared to single-object detection, our method outperforms other SOTA methods in multi-object detection. Limited by the experimental conditions, we only set the maximum value of $\varphi$ = 1 (refer to the design idea of^9^ and^27^, combined with the experimental results, it is not difficult to find that the higher the value of $\varphi$ , the better the model performance, and the higher the corresponding requirements for the experimental conditions), but even so, our method still exceeds the result of^27^ with $\varphi$ =3. The detection effect is shown in Fig. 4 (we also fuse the 2D object detection effect). Of course, whether it is 2D object detection or 6D pose estimation, we should not only consider the detection accuracy of the model but also its real-time performance. To this end, we carry out corresponding experiments.

In Table 4, considering that MagicCubePose can not only be used for 6D pose estimation but also 2D object detection, we compare our method with common object detection and pose estimation models, we not only compared with the 6D model, the 2D object detection results added too, however, considering the affect of experimental condition, we will not perform a strict horizontal comparison, we refer to the performance of some GPU and find that the performance of RTX3070 is similar to RTX2080TI, and these two methods are all based on^9^. Furthermore, to visualize the training time, we reproduce the^27^ with the same parameter $\varphi$ =0 and 2, complete training with 500 epochs of object 1 in^27^ is 2.36 and 3.86 days , it is nearly 4.4% and 70% higher than our method(2.26 days), even so, our ADD(-S) is higher. Combined with the actual test results, we can prove that our method is better, truly achieved the SOTA performance.

In general, in Table 5, we use three evaluation metrics: 5cm5degree, ADD and 2D projection to make a comprehensive and intuitive comparison of several SOTA models in the past few years. Combined with the experimental data in the previous figures and tables, it is obviously that our method is superior to the existing 6D pose estimation methods.

## Conclusion

In this paper, we introduce MagicCubePose, a 6D pose estimation model based on the extension of the 2D object detection network EfficientDet with extremely high end-to-end detection accuracy, model expansion and real-time detection performance. We adopt an intuitive and effective 2D–6D extension method similar to EfficientPose, which combines object detection, pose estimation and achieves the state-of-the-art results with superior real-time performance. In addition, our proposed method can also be applied to other 2D object detection networks and 6D pose estimation networks as a portable module or a new network structure design idea. Similarly, in future work, we hope and will apply our method to more challenging real-time tasks such as robotic grasping and autonomous driving.

We uploaded the data used in this paper to the cloud so that it could be more convenient for researchers to use. The raw data is available at the following link, it includes the train data and test results. https://drive.google.com/drive/folders/1Ah43p1yRMi2cdRfbc381a0nLUwRfMJBa?usp=share_link2.
